# Quoth the Raven: carbon monoxide and nothing more

**DOI:** 10.1186/2045-9912-3-7

**Published:** 2013-03-06

**Authors:** Leo E Otterbein

**Affiliations:** 1 Harvard Medical School and Beth Israel Deaconess Medical Center, Department of Surgery, Transplant Institute, Boston, MA, 02215, USA

## Abstract

The articles contained in this review series exemplify the diverse applications and succinct biological relevance of this simple gas. Articles summarizing the important effects of carbon monoxide in preventing the rejection of an organ, in its neuroprotective properties in piglets, regulation of mycobacterial growth, in its anti-inflammatory effects in the gut and in its use in new and innovative modalities and avenues by which to harness adjuvant therapies are eloquently and precisely described and reviewed. Each of these reports offers but a glimpse of continued prudent and sound evidence that this simple diatomic gas offers astonishingly potent and extremely diverse biological and medicinal qualities.

## 

January 1809, somewhere in Boston, Edgar Allen Poe is born, destined for literary greatness with works such as the *The Pit and the Pendulum, The Murders in the Rue Morgue* and *The Fall of the House of Usher.* Perhaps one of his more notorious works was the poem “*The Raven”* describing the pains of lost love with the black feathers indicating a black or bleak omen [1]. Edgar Allen Poe had another destiny, one ordained him to carry the prototypical outward appearance of chronic carbon monoxide poisoning thought to have been caused by leaky gas light fixtures inside his home. The symptoms included incoordination, confusion, fatigue and nausea. Perhaps most visible was the “telltale” face where his eyelid and mouth droop from partial plysis [2]. The cause of his death remains shrouded in mystery, the content of numerous theories all similarly concluding that he suffered from “congestion of the brain” due to his odd behavior when found on the streets of Baltimore in 1849. Indeed, much of his later works contain dark imagery of what has become the language associated with delusion and pnoia, symptoms that are also routinely – and interestingly - attributed to carbon monoxide poisoning. The protagonist in “*The Raven”* queries this solemn black bird, asking it questions to which the reply is always the chilling “Nevermore” [1].

Until recently, carbon monoxide was simply deemed an industrial poison, released from billowing smoke stacks along with thick, black soot, much like the downy feathers of a raven. Additionally, any conclusions regarding alternative biological properties of the gas were and continue to be, categorically classified as absurdities and therefore should be considered “Nevermore”.

The articles contained in this review series exemplify the diverse applications and succinct biological relevance of this simple gas. Articles summarizing the important effects of carbon monoxide in preventing the rejection of an organ [3], in its neuroprotective properties in piglets [4] regulation of mycobacterial growth [5] in its anti-inflammatory effects in the gut [6] and in its use in new and innovative modalities and avenues by which to harness adjuvant therapies [7] are each eloquently and precisely described and reviewed. Each of these reports offers but a glimpse of continued prudent and sound evidence that this simple diatomic gas offers astonishingly potent and extremely diverse biological and medicinal qualities. Based upon these pleiotropic effects, perhaps the gas should be defined as homeodynamic in that it befits the ever-changing requirements of the cell and tissue to ensure survival. That it does so under such distinct model systems lends credence to its importance. It could offer something beyond its being condemned as nothing but a pollutant. And, yet, despite the fact after more than a decade of research in which hundreds of published reports challenged the hardened dogma of CO’s danger, researchers find themselves in positions much like those in the past who tried to convince the world that the earth was round or that we are not the center of the universe or that one could ever fly like the Raven. Ignored, even shunned by those whose fixed beliefs, no matter how unscientifically validated, endure.

That Edgar Allen Poe suffered from the consequences of leaky furnaces is certainly possible [2], based upon canonical symptoms, but to attribute them solely to carbon monoxide poisoning is simply preposterous. No sound, well-controlled clinical study assessing the safety of carbon monoxide had been performed until recent efforts commenced to move inhaled carbon monoxide into clinical application as a therapeutic.

Indeed, recent and rigorous FDA-approved safety studies showed absolutely no untoward effects, including those that incorporated extensive neurocognitive assessments of patients with a carboxyhemoglobin level (a measure of carbon monoxide in the blood) of 12–14%; levels that historically were defined as unequivocally dangerous if not lethal.

In the 1960’s and 1970’s, a large body of research was generated describing the effects of hypoxic hypoxia in which carbon monoxide was used to create tissue hypoxia in the setting of normal perfusion. This work solidified the view that CO was a substance to be avoided. Carbon monoxide was also studied extensively in humans testing the effects of inhaled carbon monoxide on cognitive function such as memory and hand-eye coordination even having individuals drive a car during exposure. Unfortunately, most of the data was poorly controlled with varying levels of oxygen provided and vagaries on the precise regimens of CO delivered, in addition to poor controls on purity, blood levels and other critical data sets that would have made interpretation and conclusions that much more credible.

But now, nearly forty years later, FDA Phase I safety testing was unable to reproduce these long-standing findings, viz., that CO was a toxic molecule. One must ask, why? One of the fundamental and essential principles of scientific discovery is reproducibility. Clearly, this has not been observed in many of the previous studies of carbon monoxide. How have current studies resulted in such radically different conclusions from those in previous years?

Perhaps political and environmental pressures had reached their peak, those focused on limiting the rise of pollution in industries such as coal burning and automobiles? Was it less sophisticated apptus by which to generate the gas with lack of purity? Then again, the studies forty years ago in laboratories across the US were not FDA-sanctioned nor were they validated nor was there anyone postulating that the gas might offer alternative physiological effects at a tolerable concentration. Whatever the reasons, carbon monoxide was simply not being touted as a potential medical breakthrough therapy but something to be vilified. Undoubtably pressures continued to squelch any alternative conclusions that could possibly be drawn. Indeed, one opinion from a historical perspective even claims that perhaps any data that were incompatible with the current mode of thinking at the time was simply suppressed, dismissed as uninteresting artifacts.

There are currently multiple ongoing clinical trials investigating the efficacy of inhaled carbon monoxide. In 2000, the Nobel Prize in Medicine was awarded to researchers for the discovery of nitric oxide, changing forever the landscape as to how cells communicate within their environments. Small gas molecules are ideally suited as messengers able to traverse barriers and access most if not all cellular compartments. Along with carbon monoxide and nitric oxide there are other gases of great consequence and essential for life on earth like hydrogen sulfide, carbon dioxide and of course oxygen. All are potentially dangerous, but to quote the great philosopher pcelsus, “the dose makes the poison”. The same must hold true for carbon monoxide. Everything has the capability of being a poison at a set concentration even oxygen, glucose and water. Reports such as those presented in this issue of Medical Gas Research are essential and exciting additions to the field providing timely overviews in each area of focus fostering original ideas and innovative thought. At the very least they stimulate novel insight into cellular mechanics and functions that will serve to certainly advance scientific discovery.

Edgar may have succumbed to brain congestion in the end, but perhaps it was the carbon monoxide in the leaky fixture that cultivated his creativity. *The Raven* delivered its message in tones of solemn remembrance of a promise of love forever lost. Original and compelling carbon monoxide research brings a new optimism and hopefulness by many who study its protective benefits. And perhaps in the not too distant future, the condemnation of this simple gas *Shall be lifted—*for*evermore!*[1].
